# A Novel Adaptive Non-Singular Fast Terminal Sliding Mode Control for Direct Yaw Moment Control in 4WID Electric Vehicles

**DOI:** 10.3390/s25030941

**Published:** 2025-02-04

**Authors:** Jung Eun Lee, Byeong Woo Kim

**Affiliations:** Department of Electrical, Electronic and Computer Engineering, University of Ulsan, Ulsan 44610, Republic of Korea; rotnn429@ulsan.ac.kr

**Keywords:** direct yaw-moment control, yaw stability, non-singular fast terminal sliding mode control, fast reaching control law, adaptive control, 4 wheel independent drive, electric vehicle

## Abstract

This study proposes an adaptive non-singular fast terminal sliding mode control (NFTSMC)-based direct yaw moment control (DYC) strategy to enhance driving stability in four-wheel independent drive (4WID) electric vehicles. Unlike conventional SMC, the proposed method dynamically adapts to system uncertainties and reduces chattering, a critical issue in control applications. The approach begins with the development of an NFTSMC method, analyzing its performance to identify areas for improvement. To enhance robustness and responsiveness, a novel adaptive NFTSMC method is introduced. This method integrates a non-singular fast terminal sliding mode surface with a novel adaptive fast-reaching control law that combines an adaptive switching mechanism and a fast-reaching law. The designed adaptive switching law adjusts the sliding gain in real time based on system conditions, reducing chattering without needing an upper bound on uncertainties as required by traditional NFTSMC methods. Concurrently, the fast-reaching law ensures rapid convergence from any initial condition and accurate tracking performance. Simulation results across various steering maneuvers, including step, sinusoidal, and fish-hook inputs, demonstrate that the proposed method significantly improves tracking accuracy and driving stability over traditional SMC and NFTSMC methods. Marked reductions in RMS and peak yaw rate errors, and effective chattering mitigation, highlight advancements in vehicle safety and stability.

## 1. Introduction

The demand for sustainable transportation has recently accelerated the development of efficient and eco-friendly electric vehicles (EVs). EVs with four-wheel independent drive systems, in which each wheel is powered by an independent motor, offer enhanced control flexibility and energy efficiency [1]. Additionally, 4WID systems can enhance vehicle dynamic stability and steering performance by individually controlling the torque of each wheel [2]. These benefits are crucial during cornering or in situations with unstable road conditions.

Various active and passive systems, such as anti-lock brake systems (ABS) [3], active front steering (AFS) [4], autonomous emergency braking (AEB) [5,6], and direct yaw control (DYC) [2,7,8,9], have been developed to enhance vehicle safety. Among these, DYC has attracted significant attention, particularly for 4WID systems, because it leverages precise control of each wheel to maximize vehicle potential [10]. DYC improves driving stability by managing yaw moments, particularly under challenging conditions, such as sudden steering inputs or reduced traction on slippery roads at high speeds [11]. Additionally, DYC distributes torque dynamically to each wheel based on the required additional yaw moment, helping maintain the desired trajectory and effectively mitigating understeer or oversteer tendencies common in front- and rear-wheel drive vehicles.

Implementing an effective DYC system in 4WID electric vehicles poses several technical challenges. The DYC system must respond rapidly and precisely to dynamic driving conditions while maintaining vehicle stability. Inadequate control can yield excessive tire wear, reduced energy efficiency, and loss of vehicle control. Complex vehicle dynamics require robust control algorithms capable of withstanding system uncertainties, external disturbances, and modeling inaccuracies. This demand has driven the development of various control algorithms to enhance DYC performance. Among control approaches such as PID [8,12], linear quadratic regulator (LQR) [13,14], and model predictive control (MPC) [15,16], sliding mode control (SMC) [2,7,17,18,19] is considered suitable for DYC owing to its robustness and relatively simple implementation.

Traditional SMC employs a linear sliding surface that provides asymptotic stability. However, it lacks finite-time convergence, which can lead to prolonged tracking error persistence. Terminal SMC (TSMC) [20,21] was developed, enabling finite-time convergence to achieve a faster response to address this limitation. However, TSMC has limitations too, such as variability in convergence speed depending on the initial system state relative to the equilibrium point and potential singularity problems. Consequently, fast terminal SMC (FTSMC) and non-singular terminal SMC (NTSMC) were introduced to increase convergence speed and eliminate singularities. Despite these enhancements, all these methods focus on isolated issues rather than providing a holistic solution. Therefore, non-singular fast terminal SMC (NFTSMC) [2,7,22] was proposed, offering rapid convergence and robust, stable control while mitigating singularity risks.

The chattering phenomenon in control signals is an inherent issue in SMC-based controllers, including TSMC and its variants (FTSMC, NTSMC, and NFTSMC), due to the discontinuous nature of the control law [23]. To mitigate this problem, several advanced techniques have been developed. Higher-order SMC methods [24] smooth the control signal by incorporating additional derivatives of the sliding variable. Neural networks [2,7,17,20,25] adaptively estimate and compensate for system uncertainties in real time. Fuzzy logic control [26] reduces reliance on abrupt switching by employing linguistic rules. Boundary layer techniques [27] smooth the discontinuous sign function to suppress chattering. Disturbance observers [21,28] minimize the impact of external disturbances. Additionally, super-twisting control [29] enhances robustness by reducing chattering through a continuous sliding mode, without requiring switching. These methods, each addressing different aspects of chattering, have shown promising results in improving the stability and robustness of control systems in various applications, including electric vehicle dynamics. Each approach offers trade-offs regarding its robustness and implementation complexity. Recently, adaptive control techniques [17,30] have been introduced to provide a comprehensive solution by dynamically adjusting the switching gain according to the system state and control error. By increasing the sliding gain when the control error is large to maintain robustness and reducing it when the error is small to minimize chattering, adaptive control effectively balances robustness with reduced chattering, which is key for maintaining precise and stable control.

A high convergence speed is crucial for accommodating diverse driving conditions, from low-speed maneuvers to high-speed responses, and for rapidly countering disturbances such as abrupt steering or changes in the road surface. Therefore, recent advancements have focused on enhanced reaching laws to address this. In particular, the super-fast reaching control law accelerates the system’s approach to the sliding surface, ensuring rapid stabilization in time-sensitive scenarios. This improvement significantly enhances the system’s ability to adapt to sudden changes in driving conditions [25,31].

Expanding on previous studies, this study proposes a novel control strategy based on NFTSMC for the DYC of 4WID EVs. This approach enhances control accuracy and convergence speed while addressing the singularity issues inherent in traditional SMC. The switching gain is dynamically adjusted by integrating adaptive control and a fast reaching law, addressing the challenges of chattering, convergence speed, and robustness holistically. The main contributions of this study are as follows:

- An innovative NFTSMC method for the DYC system: This study introduces a novel NFTSMC-based control framework specifically designed for the DYC system of 4WID EVs. By integrating advanced control strategies, the proposed NFTSMC method achieves a robust and flexible control structure, ensuring enhanced system stability, rapid adaptation to dynamic driving conditions, and the effective mitigation of nonlinearities and disturbances.
- Enhanced convergence and improved yaw rate tracking accuracy: The combination of NFTSMC and the adaptive super-fast reaching control law facilitates high-speed convergence to the sliding surface while maintaining precise yaw rate tracking. This enhancement not only addresses the singularity issues inherent to traditional methods but also ensures superior responsiveness across diverse driving scenarios, including sudden steering inputs.
- Superior chattering mitigation: The proposed adaptive control mechanism dynamically adjusts the sliding gain based on the system state condition within the reaching control law, effectively counteracting nonlinearities and uncertainties introduced by abrupt disturbances in the vehicle’s complex dynamics. This approach minimizes or eliminates chattering in the control input due to external disturbances and system uncertainties, enhancing robustness against system parameter variations and improving overall control performance to ensure safe and stable operation.
- Eliminating prior knowledge of disturbance: Accurately determining the upper boundary is challenging in real-world applications. This adaptive sliding gain dynamically changes based on the state of the sliding surface, thereby enhancing system performance by eliminating the need for precise knowledge of the upper boundary of uncertainties. This approach improves the practical applicability of the control strategy and simplifies its implementation in real-world driving environments.
- Rigorous theoretical stability verification using Lyapunov theory: The stability of the proposed NFTSMC method was rigorously validated using Lyapunov theory, providing a solid theoretical foundation for its robustness and reliability.
- Comprehensive validation: Extensive simulations were performed using CarSim and Matlab under various driving scenarios. The results demonstrate significant improvements in tracking accuracy and convergence speed and a substantial reduction in control signal chattering, validating the effectiveness of the proposed control strategy.

The remainder of this study is organized as follows: Section 2 outlines the vehicle dynamics model and research problems utilized in the design of the DYC system. Section 3 discusses the design process of the DYC system, applying the proposed innovative NFTSMC method. Section 4 presents the simulation environment and results and analyzes the performance of the proposed control strategy. Finally, Section 5 summarizes the research findings and discusses future research directions.

## 2. Problem Statement

This section discusses the vehicle dynamics model and formulates the control problem addressed in this study. A 7-degree-of-freedom (7-DOF) nonlinear vehicle dynamics model is employed to design a control strategy with additional yaw moment control at the upper controller level. This model is essential for validating the proposed hierarchical control framework.

### 2.1. Description of Vehicle Dynamic Model

**Figure 1:**
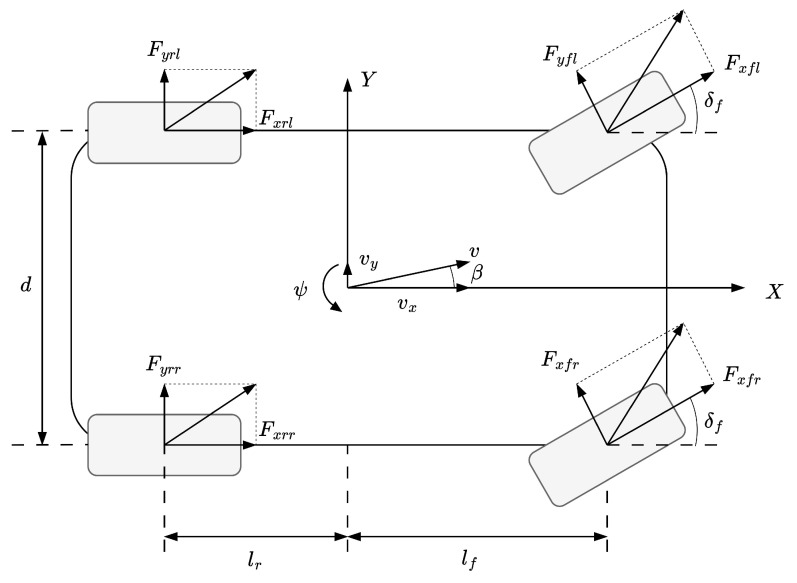
7-DOF vehicle dynamic model.

Figure 1 illustrates the 7-DOF vehicle dynamics model utilized in this study. The 7-DOF model is specifically adopted for control design purposes to address the complexities of vehicle lateral dynamics. It incorporates the primary dynamics required to calculate the control inputs while maintaining a balance between accuracy and computational efficiency.

To simplify the model and focus on the primary dynamics, the following assumptions are applied:

- The two front wheels share an identical steering angle.
- All tires have zero camber angles.
- Tire self-alignment moments are considered negligible.
- The roll axis inclination angle relative to the horizontal is minimal and not essential.
- Additional steering angles due to compliance and roll steer effects are negligible.
- All products of inertia are neglected.

This model characterizes longitudinal, lateral, and yaw motions, along with the rotational dynamics of the four wheels as follows [9]:

(1) $$\left\{\begin{array}{c} ma_{x}=m\left({\dot{v}}_{x}-v_{x}\gamma \right)=F_{x}\\ ma_{y}=m\left({\dot{v}}_{y}+v_{x}\gamma \right)=F_{y}\\ I_{z}\dot{\gamma }=M_{z} \end{array}\right.$$

The right-hand terms of Equation (1) are defined as

1. Longitudinal motion:
  (2) $$F_{x}=F_{xrl}+F_{xrr}-\left(F_{yfl}+F_{yfr}\right)\sin\delta_{f}+\left(F_{xfl}+F_{xfr}\right)\cos\delta_{f}$$
2. Lateral motion:
  (3) $$F_{y}=\left(F_{yfl}+F_{yfr}\right)\cos\delta_{f}-\left(F_{xfl}+F_{xfr}\right)\sin\delta_{f}+F_{yrl}+F_{yrr}$$
3. Yaw motion:
  (4) $$\begin{array}{cc} M_{z} & =l_{f}\left[\left(F_{xfl}+F_{xfr}\right)\sin\delta_{f}+\left(F_{yfl}+F_{yfr}\right)\cos\delta_{f}\right]-l_{r}\left(F_{yrl}+F_{yrr}\right)\\ \; & \;+\frac{d_{f}}{2}\left[\left(F_{xfr}-F_{xfl}\right)\cos\delta_{f}+\left(F_{yfl}-F_{yfr}\right)\sin\delta_{f}\right]+\frac{d_{f}}{2}\left(F_{xrr}-F_{xrl}\right) \end{array}$$

where *m* denotes the vehicle mass, $v_{x}$ and $v_{y}$ are the longitudinal and lateral velocities, and $a_{x}$ and $a_{y}$ represent longitudinal and lateral accelerations. Variables γ, $I_{z}$, and $\delta_{f}$ denote the yaw rate, yaw inertia, and front wheel steering angle, respectively. $l_{f}$ and $l_{r}$ are the distances from the vehicle center to the front and rear axles, while $d_{f}$ and $d_{r}$ denote the front and rear wheel tracks. Force terms $F_{xfl}$, $F_{xfr}$, $F_{xrl}$, and $F_{xrr}$ represent longitudinal forces on each tire, and $F_{yfl}$, $F_{yfr}$, $F_{yrl}$, and $F_{yrr}$ denote lateral forces on each tire. Lastly, $M_{z}$ is the yaw moment.

### 2.2. Problem Formulation

Given the 4WID electric vehicle model, additional direct yaw moments can be generated by the electric motors on each wheel to maintain lateral stability. The vehicle’s yaw dynamics can be expressed as follows:

(5) $$\begin{array}{cc} M_{z} & =l_{f}\left[\left(F_{xfl}+F_{xfr}\right)\sin\delta_{f}+\left(F_{yfl}+F_{yfr}\right)\cos\delta_{f}\right]-l_{r}\left(F_{yrl}+F_{yrr}\right)\\ \; & \;+\frac{d_{f}}{2}\left[\left(F_{xfr}-F_{xfl}\right)\cos\delta_{f}+\left(F_{yfl}-F_{yfr}\right)\sin\delta_{f}\right]+\frac{d_{f}}{2}\left(F_{xrr}-F_{xrl}\right)+\Delta M_{z}+D \end{array}$$

where $\Delta M_{z}$ denotes the additional yaw moment generated by the controller, and *D* represents uncertainties and external disturbances, bounded by $\left|D\right|\leq \bar{D}$, with $\bar{D}>0$.

**Figure 2:**
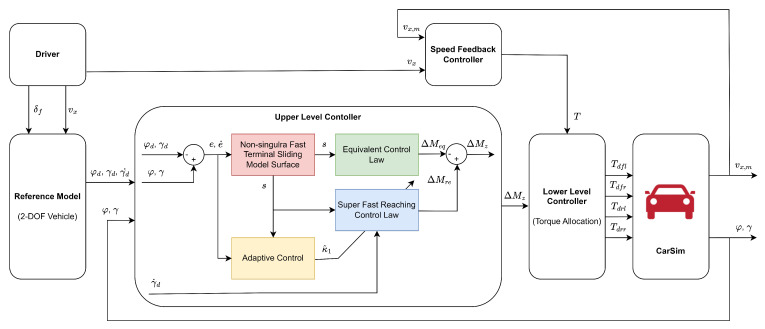
Architecture of the proposed system.

Figure 2 illustrates a hierarchical structure adopted to compute the additional torque required at each wheel and address this control problem. The proposed architecture includes a reference model and upper and lower controllers. The reference model, which utilizes a 2-DOF vehicle dynamics model, provides a target yaw rate that represents the desired vehicle behavior. Using the reference model as a foundation, the 7-DOF vehicle model serves as a dynamic model for designing the upper controller using the proposed NFTSMC approach. This upper controller calculates the additional yaw moment $\Delta M_{z}$ to enhance lateral stability by minimizing the yaw rate error. Subsequently, the lower controller spreads this additional yaw moment across each wheel through a static distribution algorithm. This study aims to design a stability controller that enhances lateral stability and ensures precise tracking of the reference yaw rate.

## 3. Design of Control System

### 3.1. Reference Model

To achieve vehicle driving stability, the control system employs a reference model tracking strategy. The reference yaw rate serves as the target response that the vehicle must follow because it is a fundamental measure of vehicle handling performance.

In this study, a linear 2-DOF vehicle dynamics model is used to generate the reference response. The model is constructed based on the assumption that the wheel slip angles are small and that the longitudinal velocity at the vehicle’s center is constant. Such simplifications enable the model to effectively capture the relationship between the steering input and the yaw rate, ensuring its suitability for control purposes.

**Figure 3:**
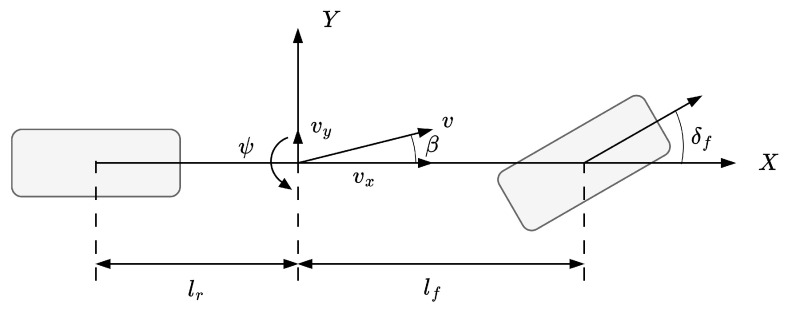
2-DOF vehicle dynamic model.

The 2-DOF model plays a critical role in the control architecture by providing a computationally efficient way to approximate the desired yaw rate. This approximation reduces the computational burden while maintaining sufficient accuracy for the stability control design. Figure 3 illustrates the 2-DOF model used in this analysis.

The equations governing the lateral and yaw dynamics of the 2-DOF model are expressed as below [2]:

(6) $$\left\{\begin{array}{c} m\left(\dot{v_{y}}+v_{x}\gamma \right)=\left(k_{f}+k_{r}\right)\beta +\frac{1}{v_{x}}\left(l_{f}k_{f}-l_{r}k_{r}\right)\gamma -k_{f}\delta_{f}\\ I_{z}\dot{\gamma }=\left(l_{f}k_{f}-l_{r}k_{r}\right)\beta +\frac{1}{v_{x}}\left(l_{f}^{2}k_{f}-l_{r}^{2}k_{r}\right)\gamma -l_{f}k_{f}\delta_{f} \end{array}\right.$$

where γ represents the yaw rate, β denotes the sideslip angle, and $k_{f}$ and $k_{r}$ are the cornering stiffness coefficients of the front and rear wheels, respectively.

For a very small sideslip angle, it can be approximated as $\beta =\tan^{-1}\left(\frac{v_{y}}{v_{x}}\right)\approx \frac{v_{y}}{v_{x}}$. Using this approximation, the 2-DOF model can be reformulated as follows:

(7) $$\left\{\begin{array}{c} \dot{\beta }=\frac{k_{f}+k_{r}}{mv_{x}}\beta +\left(\frac{l_{f}k_{f}-l_{r}k_{r}}{mv_{x}^{2}}-1\right)\gamma -\frac{k_{f}}{mv_{x}}\delta_{f}\\ \dot{\gamma }=\frac{l_{f}k_{f}-l_{r}k_{r}}{I_{z}}\beta -\frac{l_{f}k_{f}}{I_{z}}\delta_{f}+\frac{{l_{f}}^{2}k_{f}+{l_{r}}^{2}k_{r}}{I_{z}v_{x}}\gamma \end{array}\right.$$

For steady-state conditions, where γ is assumed to be constant, the desired steady-state yaw rate can be calculated as

(8) $$\gamma_{s}=\frac{v_{x}}{L\left(1+Kv_{x}^{2}\right)}\delta_{f}$$

where $K=\frac{m}{L^{2}}\left(\frac{l_{f}}{k_{f}}-\frac{l_{r}}{k_{r}}\right)$ represents the stability factor for the steady-state response of a vehicle.

To ensure that the reference yaw rate does not yield excessive lateral acceleration beyond tire adhesion limits, the influence of road surface friction is included [23]. Therefore, the final reference yaw rate is defined as follow:

(9) $$\gamma_{d}=\left\{\begin{array}{cc} \gamma_{s} & \left|\gamma_{s}\right|<0.85\frac{\mu g}{v_{x}}\\ 0.85\frac{\mu g}{v_{x}}\mathrm{sgn}\left(\gamma_{s}\right) & \left|\gamma_{s}\right|\geq 0.85\frac{\mu g}{v_{x}} \end{array}\right.$$

where μ represents the road surface friction coefficient, and *g* is the gravitational acceleration.

### 3.2. Upper Level Controller

#### 3.2.1. Design of the Non-Singular Fast Terminal SMC

To design the upper-level controller, the yaw angle and yaw rate errors are first defined as [2]:

(10) $$e=\psi -\psi_{d},\;\dot{e}=\gamma -\gamma_{d}$$

where $\psi_{d}$ and $\gamma_{d}$ denote the desired yaw angle and yaw rate, respectively.

Next, the NFTSM surface is constructed to ensure rapid error convergence without singularity:

(11) $$s=e+\lambda_{1}{\left|e\right|}^{p}\mathrm{sgn}\left(e\right)+\lambda_{2}{\left|\dot{e}\right|}^{q}\mathrm{sgn}\left(\dot{e}\right)$$

where $\lambda_{1}$ and $\lambda_{2}$ are positive constants, and *p* and *q* must satisfy the conditions 1<q<2, p>q. These values can be selected by the user to adjust the shape of the NFTSM surface.

After the reaching phase, the sliding condition s=0 is achieved. From Equation (11), this implies:

(12) $$0=e+\lambda_{1}{\left|e\right|}^{p}\mathrm{sgn}\left(e\right)+\lambda_{2}{\left|\dot{e}\right|}^{q}\mathrm{sgn}\left(\dot{e}\right)$$

which can be rewritten as:

(13) $$\left(\left|e\right|+\lambda_{1}{\left|e\right|}^{p}\right)\mathrm{sgn}\left(e\right)=-\lambda_{2}{\left|\dot{e}\right|}^{q}\mathrm{sgn}\left(\dot{e}\right)$$

From this, we derive the relationship:

(14) $$\left(\left|e\right|+\lambda_{1}{\left|e\right|}^{p}\right)=\lambda_{2}{\left|\dot{e}\right|}^{q},\;\mathrm{sgn}\left(e\right)=-\mathrm{sgn}\left(\dot{e}\right)$$

Hence,

(15) $$|\dot{e}|={\left(\frac{1}{\lambda_{2}}\left|e\right|+\frac{\lambda_{1}}{\lambda_{2}}{\left|e\right|}^{p}\right)}^{\frac{1}{q}}$$

Substituting this into $\dot{e}$, we get:

(16) $$\dot{e}=\left|\dot{e}\right|\mathrm{sgn}\left(\dot{e}\right)=-{\left(\frac{1}{\lambda_{2}}\left|e\right|+\frac{\lambda_{1}}{\lambda_{2}}{\left|e\right|}^{p}\right)}^{\frac{1}{q}}\mathrm{sgn}\left(e\right)$$

Now, consider the Lyapunov function $V=0.5e^{2}$. Its time derivative is:

(17) $$\dot{V}=e\dot{e}=-e{\left(\frac{1}{\lambda_{2}}\left|e\right|+\frac{\lambda_{1}}{\lambda_{2}}{\left|e\right|}^{p}\right)}^{\frac{1}{q}}\mathrm{sgn}\left(e\right)$$

Simplifying further, we obtain:

(18) $$\dot{V}=-{\left(\frac{1}{\lambda_{2}}{\left(2V\right)}^{\frac{1+q}{2}}+\frac{\lambda_{1}}{\lambda_{2}}{\left(2V\right)}^{\frac{p+q}{2}}\right)}^{\frac{1}{q}}$$

This can be expressed as:

(19) $$\dot{V}=-{\left(a_{1}V^{b_{1}}+a_{2}V^{b_{2}}\right)}^{\varpi }$$

where

$$a_{1}=\frac{1}{\lambda_{2}}2^{\frac{1+q}{2}},\;a_{2}=\frac{\lambda_{1}}{\lambda_{2}}2^{\frac{p+q}{2}},\;b_{1}=\frac{1+q}{2},\;b_{2}=\frac{p+q}{2},\;\varpi =\frac{1}{q}.$$

With the choice 1<q<2 and p>q, we ensure $0.5<b_{1}\varpi <1$ and $b_{2}\varpi >1$. These conditions align with the fixed-time convergence structure discussed in [32,33]. Consequently, during the sliding phase, the tracking error *e* converges to zero within a fixed time.

**Remark** **1.** *The sliding surface defined in Equation (11) using the NFTSM concept is advantageous for control system performance. One key feature is its ability to converge rapidly to the equilibrium point whether the initial state is near or far from it. This characteristic is crucial for systems that must respond quickly to varying conditions. Moreover, this surface effectively addresses the singularity issue common in traditional TSMC, where the control action can become undefined when e=0 and $\dot{e}=0$. By overcoming this limitation, the designed surface ensures that control remains continuous and well-defined under all conditions, thus enhancing the system’s reliability and robustness. This property makes it well suited to applications where rapid convergence and the avoidance of singularities are essential for stable operation. The aforementioned sliding surface is utilized in this study for its capability to improve overall system stability and performance.*

The vehicle’s yaw motion dynamics, based on Equation (5), can be expressed as

(20) $$\begin{array}{cc} \dot{\gamma } & =\frac{1}{I_{z}}\left[l_{f}\left(F_{yfl}+F_{yfr}\right)\cos\delta_{f}+\frac{d_{f}}{2}\left(F_{yfl}-F_{yfr}\right)\sin\delta_{f}-l_{r}\left(F_{yrl}+F_{yrr}\right)+\Delta M_{z}+D\right]\\ \; & =\frac{1}{I_{z}}\left(F+\Delta M_{z}+D\right) \end{array}$$

where *F* represents the combined effect of tire forces. It is defined as

(21) $$F=l_{f}\left(F_{yfl}+F_{yfr}\right)\cos\delta_{f}+\frac{d_{f}}{2}\left(F_{yfl}-F_{yfr}\right)\sin\delta_{f}\;-l_{r}\left(F_{yrl}+F_{yrr}\right)$$

By combining Equations (10) and (20), the derivative of the sliding surface is derived as

(22) $$\dot{s}=\dot{e}+p\lambda_{1}{\left|e\right|}^{p-1}\dot{e}+q\lambda_{2}{\left|\dot{e}\right|}^{q-1}\left(\frac{1}{I_{z}}\left(F+\Delta M_{z}+D\right)-\dot{\gamma_{d}}\right)$$

Based on Equation (22), the NFTSMC law is designed as

(23) $$\left\{\begin{array}{c} \Delta M_{z}=\Delta M_{eq}+\Delta M_{r}\\ \Delta M_{eq}=I_{z}\left[\dot{\gamma_{d}}-\frac{{\left|\dot{e}\right|}^{2-q}}{q\lambda_{2}}\left(1+p\lambda_{1}{\left|e\right|}^{p-1}\right)\mathrm{sgn}\left(\dot{e}\right)\right]-F\\ \Delta M_{r}=-I_{z}\left[\left(\kappa_{1}+\vartheta \right)\mathrm{sgn}\left(s\right)\right] \end{array}\right.$$

where $\Delta M_{eq}$ is the equivalent control law, $\Delta M_{r}$ is the reaching control law, $\kappa_{1}=\bar{D}/I_{z}$ represents the upper bound of uncertainties, and ϑ is a small positive constant.

The stability of the NFTSMC system is analyzed as follows:

Substituting the control law in Equation (23) into Equation (22), we obtain:

(24) $$\begin{array}{cc} \dot{s} & =q\lambda_{2}{\left|\dot{e}\right|}^{q-1}\left(\frac{1}{I_{z}}\left(-I_{z}\left[\left(\kappa_{1}+\vartheta \right)\mathrm{sgn}\left(s\right)\right]+D\right)\right)\\ \; & =A\left(\frac{D}{I_{z}}-\left(\kappa_{1}+\vartheta \right)\mathrm{sgn}\left(s\right)\right) \end{array}$$

where $A=q\lambda_{2}{\left|\dot{e}\right|}^{q-1}>0$ for all $\dot{e}$ is not equal 0 and it can bounded by $A\leq \bar{A}$.

Choosing the Lyapunov function $V_{1}=0.5s^{2}$, its time derivative is given by

(25) $$\begin{array}{cc} {\dot{V}}_{1} & =s\dot{s}\\ \; & =sA\left(\frac{D}{I_{z}}-\left(\kappa_{1}+\vartheta \right)\mathrm{sgn}\left(s\right)\right)\\ \; & =A\left[\left(\frac{Ds}{I_{z}}-\kappa_{1}\left|s\right|\right)-\vartheta \left|s\right|\right]\\ \; & \leq A\left[\left(\frac{\bar{D}}{I_{z}}-\kappa_{1}\right)\left|s\right|-\vartheta |s|\right]\\ \; & \leq -A\vartheta |s|<0 \end{array}$$

The result found using Equation (25) shows that $V_{1}>0$ and ${\dot{V}}_{1}<0$, indicating that the sliding variable *s* will converge to zero and the tracking error *e* will converge to the equilibrium point.

To ensure the stability of the control system, the sliding gain $\left(\kappa_{1}+\vartheta \right)$ must exceed the maximum value of the system’s uncertainty components. However, accurately determining this upper boundary is challenging in real-world applications. If the sliding gain is set too high, it results in significant chattering, rapid, oscillatory control actions that can damage mechanical components and reduce system efficiency. This is highly undesirable in practical applications. Additionally, the conventional control law in Equation (23) may yield a slow convergence of *s* to zero, which limits the system’s responsiveness.

Therefore, we propose an adaptive reach control law for the NFTSMC that incorporates a fast-reaching law and an adaptive mechanism for adjusting the sliding gain to address these challenges. This adaptive sliding gain dynamically changes based on the state of *s*, thereby enhancing system performance by reducing chattering and eliminating the need for precise knowledge of the upper boundary of uncertainties. The fast-reaching law ensures a quicker convergence of *s* to zero, improving the overall response speed of the system. These features yield a more robust and efficient control system capable of maintaining stability without the drawbacks of excessive fixed gains.

#### 3.2.2. Design of Novel Adaptive Non-Singular Fast Terminal SMC

The proposed adaptive NFTSMC law is formulated as

(26) $$\left\{\begin{array}{c} \Delta M_{z}=\Delta M_{eq}+\Delta M_{ar}\\ \Delta M_{eq}=I_{z}\left[\dot{\gamma_{d}}-\frac{{\left|\dot{e}\right|}^{2-q}}{q\lambda_{2}}\left(1+p\lambda_{1}{\left|e\right|}^{p-1}\right)\mathrm{sgn}\left(\dot{e}\right)\right]-F\\ \Delta M_{ar}=-I_{z}\left[{\hat{\kappa }}_{1}\mathrm{sgn}\left(s\right)+\frac{1}{N\left(s\right)}\left(\kappa_{2}{\mathrm{sig}}^{r_{1}}\left(s\right)+\kappa_{3}{\mathrm{sig}}^{r_{2}}\left(s\right)\right)\right] \end{array}\right.$$

where $\Delta M_{eq}$ represents the equivalent control law as in Equation (23), while $\Delta M_{ar}$ is the novel adaptive fast-reaching control law. This adaptive law combines an adaptive sliding gain ${\hat{\kappa }}_{1}$ with a super-fast reaching law [25,31]. $N\left(s\right)=\epsilon +\left(1-\epsilon \right)\exp\left(-n{\left|s\right|}^{m}\right)$, 0<ϵ<1, n>0, *m* is an even integer, $\kappa_{2}>0$, $\kappa_{3}>0$, $r_{1}=l_{1}^{\mathrm{sgn}\left(\left|s\right|-1\right)}$, $r_{2}=l_{2}^{\mathrm{sgn}\left(1-\left|s\right|\right)}$, $l_{1}>1$, and $0.5<l_{2}<1$.

The sliding gain’s adaptive rule is specified as [30,34]:

(27) $${\dot{\hat{\kappa }}}_{1}=\left\{\begin{array}{cc} A\frac{1}{\rho }\left|s\right| & \mathrm{if}\;\left|s\right|\geq \upsilon \\ -G^{-1}{\hat{\kappa }}_{1}\frac{\left|s\right|}{\upsilon } & \mathrm{if}\;\left|s\right|<\upsilon \end{array}\right.$$

where ρ>0 and υ>0 are positive constants, and G>0 is a sampling time constant.

**Remark** **2.** *The adaptive rule for the sliding gain in Equation (27) dynamically adjusts ${\hat{\kappa }}_{1}$ based on the system state, ensuring effective control performance while minimizing chattering. Specifically:*

- *When |s| exceeds the threshold value υ, the sliding gain ${\hat{\kappa }}_{1}$ rapidly increases. This ensures a sufficient control effort to drive s toward the sliding surface, enabling quick convergence to a region where |s|<υ.*
- *Once |s| enters this smaller region (indicating minimal error), the adaptive rule gradually decreases ${\hat{\kappa }}_{1}$, reducing unnecessary control efforts. This mitigates the high-frequency switching typical in fixed-gain sliding mode controllers, significantly reducing chattering and enhancing system performance.*

*Additionally, by incorporating the current value of the sliding gain ${\hat{\kappa }}_{1}$ within the adaptive rule, ${\hat{\kappa }}_{1}$ is ensured to remain positive. When |s|<υ, the term $-G^{-1}{\hat{\kappa }}_{1}\frac{\left|s\right|}{\upsilon }$ decreases ${\hat{\kappa }}_{1}$ without allowing it to become negative. This is attributable to the decay term being directly proportional to ${\hat{\kappa }}_{1}$. As ${\hat{\kappa }}_{1}$ approaches zero, the decay rate decreases, preventing ${\hat{\kappa }}_{1}$ from turning negative. This adaptive mechanism ensures a consistently positive ${\hat{\kappa }}_{1}$, crucial for system stability and reliable control performance.*

**Remark** **3.** *The fast-reaching law $\Delta M_{ar}=\frac{1}{N(s)}\left(\kappa_{2}{\mathrm{sig}}^{r_{1}}\left(s\right)+\kappa_{3}{\mathrm{sig}}^{r_{2}}\left(s\right)\right)$ facilitates rapid convergence of the sliding surface s to zero, despite the initial conditions. When |s|>1, the function N(s) decreases, increasing $\frac{1}{N(s)}$. This amplification, combined with suitable updated exponent coefficients, where $r_{1}=l_{1}>1$ and $r_{2}=1/l_{2}>1$, effectively propels s toward one with a greater speed. Conversely, as |s|<1 approaches zero, N(s) approaches one, enabling the system to maintain a stable control action. In this range, the exponent coefficients are adjusted to $r_{1}=1/l_{1}<1$ and $r_{2}=l_{2}<1$, which facilitates the swift convergence of s to zero. This dynamic adjustment of N(s), $r_{1}$, and $r_{2}$ is crucial in ensuring rapid and robust convergence of the sliding surface to the desired equilibrium point. By adopting these parameters based on the current state of s, the controller can effectively manage its convergence behavior, maintaining system stability and performance across various operational conditions.*

**Remark** **4.** *The parameters of the proposed control method are selected based on the guidelines outlined in this paper. To assist in their selection, we provide a detailed explanation of how these parameters influence the performance of this control method:*

- *$\lambda_{1},\lambda_{2},\kappa_{2},\kappa_{3}$: Increasing these parameters enhances the system’s robustness to uncertainties and external disturbances while improving its convergence properties.*
- *p and $l_{1}$: Larger values of these parameters enable faster stabilization when the system state exceeds a predefined threshold, accelerating the convergence process. This adjustment is particularly effective in handling large deviations.*
- *q and $l_{2}$: These parameters primarily affect the system’s behavior when the state remains close to the equilibrium point (i.e., within a small bound). Adjusting them can improve precision and fine-tune the system’s control performance to near the desired state.*
- *υ: The threshold for the adaptive parameter $\kappa_{1}$ plays a critical role in balancing stability and responsiveness:*
  – *A higher υ reduces sensitivity to minor state changes, minimizing chattering and enhancing robustness against noise. However, it may result in slower response times and reduced precision.*
  – *A lower υ increases responsiveness and accuracy but can make the system more sensitive to disturbances or noise.*
*For systems subject to high noise or frequent disturbances, a higher υ is preferable to avoid overreactive adjustments. In contrast, systems requiring rapid responses benefit from a lower υ, which facilitates quicker adaptation.*
- *ρ: This parameter inversely affects the adaptation rate of $\kappa_{1}$. Smaller ρ values increase the system’s responsiveness to larger deviations, potentially accelerating the convergence process, while larger ρ values slow the rate of adaptation, improving stability and noise tolerance.*

*To achieve optimal performance, the parameters should be fine-tuned iteratively through a combination of trial-and-error testing and performance evaluations. This approach ensures that the control method is tailored to meet the specific demands of the system, balancing robustness, responsiveness, and precision under various operating conditions.*

#### 3.2.3. Stability Verification of Proposed Controller

By substituting the proposed control law from Equation (26) into Equation (22), we obtain:

(28) $$\dot{s}=A\left[\frac{D}{I_{z}}-{\hat{\kappa }}_{1}\mathrm{sgn}\left(s\right)-\frac{1}{N(s)}\left(\kappa_{2}{\mathrm{sig}}^{r_{1}}\left(s\right)+\kappa_{3}{\mathrm{sig}}^{r_{2}}\left(s\right)\right)\right]$$

To analyze stability, we define the following Lyapunov function:

(29) $$V_{2}=\frac{1}{2}s^{2}+\frac{1}{2}\rho {\tilde{\kappa }}_{1}^{2}$$

where ${\tilde{\kappa }}_{1}=\kappa_{1}-{\hat{\kappa }}_{1}$ represents the estimation error of ${\hat{\kappa }}_{1}$.

Taking the time derivative of $V_{2}$ and substituting $\dot{s}$ from Equation (28), we get:

(30) $$\begin{array}{cc} {\dot{V}}_{2} & =s\dot{s}+\rho {\tilde{\kappa }}_{1}{\dot{\tilde{\kappa }}}_{1}\\ \; & =s\dot{s}-\rho {\tilde{\kappa }}_{1}{\hat{\kappa }}_{1}\\ \; & =s\left[A\left(\frac{D}{I_{z}}-{\hat{\kappa }}_{1}\;\mathrm{sgn}\left(s\right)-\frac{1}{N(s)}\left(\kappa_{2}{\mathrm{sig}}^{r_{1}}\left(s\right)+\kappa_{3}{\mathrm{sig}}^{r_{2}}\left(s\right)\right)\right)\right]-\rho \left(\kappa_{1}-{\hat{\kappa }}_{1}\right){\dot{\hat{\kappa }}}_{1} \end{array}$$

We consider two cases depending on the sliding gain condition due to adaptive control as follows:

**Case 1:** When |s|≥υ

For |s|≥υ, ${\dot{\hat{\kappa }}}_{1}=A\frac{1}{\rho }\left|s\right|$. Here, ${\dot{V}}_{2}$ becomes

(31) $$\begin{array}{cc} {\dot{V}}_{2} & =s\left(A\frac{D}{I_{z}}-A{\hat{\kappa }}_{1}\;\mathrm{sgn}\left(s\right)-\frac{A}{N(s)}\left(\kappa_{2}{\mathrm{sig}}^{r_{1}}\left(s\right)+\kappa_{3}{\mathrm{sig}}^{r_{2}}\left(s\right)\right)\right)-\rho \left(\kappa_{1}-{\hat{\kappa }}_{1}\right)A\frac{\left|s\right|}{\rho }\\ \; & =sA\frac{D}{I_{z}}-A{\hat{\kappa }}_{1}\left|s\right|-A\left(\kappa_{1}-{\hat{\kappa }}_{1}\right)\left|s\right|-A\left[\frac{1}{N(s)}\left(\kappa_{2}{\left|s\right|}^{r_{1}+1}+\kappa_{3}{\left|s\right|}^{r_{2}+1}\right)\right]\\ \; & \leq A\left(\frac{\bar{D}}{I_{z}}-\kappa_{1}\right)\left|s\right|-A\left[\frac{1}{N(s)}\left(\kappa_{2}{\left|s\right|}^{r_{1}+1}+\kappa_{3}{\left|s\right|}^{r_{2}+1}\right)\right]\\ \; & \leq -A\left[\frac{1}{N(s)}\left(\kappa_{2}{\left|s\right|}^{r_{1}+1}+\kappa_{3}{\left|s\right|}^{r_{2}+1}\right)\right]\\ \; & \leq -A\left(\kappa_{2}{\left|s\right|}^{r_{1}+1}+\kappa_{3}{\left|s\right|}^{r_{2}+1}\right)<0 \end{array}$$

Since ${\dot{V}}_{2}$ is negative definite, the sliding variable *s* and the gain estimation error ${\tilde{\kappa }}_{1}$ are both bounded. To demonstrate the rapid convergence of *s* to within υ, we introduce a new Lyapunov function $V_{3}=s^{2}$, whose time derivative is:

(32) $${\dot{V}}_{3}=2s\dot{s}$$

Substituting the dynamics of $\dot{s}$, we obtain:

(33) $$\begin{array}{cc} {\dot{V}}_{3} & =2sA\left[\frac{D}{I_{z}}-{\hat{\kappa }}_{1}\mathrm{sgn}\left(s\right)-\frac{1}{N(s)}\left(\kappa_{2}{\mathrm{sig}}^{r_{1}}\left(s\right)+\kappa_{3}{\mathrm{sig}}^{r_{2}}\left(s\right)\right)\right]\\ \; & \leq 2A\left[\left(\frac{\bar{D}}{I_{z}}-{\hat{\kappa }}_{1}\right)\left|s\right|-\frac{1}{N(s)}\left(\kappa_{2}{\left|s\right|}^{r_{1}+1}+\kappa_{3}{\left|s\right|}^{r_{2}+1}\right)\right]\\ \; & \leq 2A\left[{\tilde{\kappa }}_{1}\left|s\right|-\left(\kappa_{2}{\left|s\right|}^{r_{1}+1}+\kappa_{3}{\left|s\right|}^{r_{2}+1}\right)\right]\\ \; & \leq -2A\left(\kappa_{2}{\left|s\right|}^{r_{1}+1}+\kappa_{3}{\left|s\right|}^{r_{2}+1}\right)+2A{\tilde{\kappa }}_{1}\left|s\right| \end{array}$$

Letting ${\bar{\kappa }}_{2}=2A\kappa_{2}$, ${\bar{\kappa }}_{3}=2A\kappa_{3}$, and $\ell =2A{\tilde{\kappa }}_{1}\left|s\right|$, we rewrite this as:

(34) $${\dot{V}}_{3}\leq -{\bar{\kappa }}_{2}\left(V_{3}^{\frac{r_{1}+1}{2}}\right)-{\bar{\kappa }}_{3}\left(V_{3}^{\frac{r_{2}+1}{2}}\right)+\ell$$

From earlier analysis, it was established that *s* and ${\tilde{\kappa }}_{1}$ are bounded, ensuring *ℓ* remains bounded. We further consider two cases:

- When |s|>1:
  For $r_{1}=l_{1}>1$ and $r_{2}=1/l_{2}>1$, it can be shown that:
  $$-{\bar{\kappa }}_{2}V_{3}^{\frac{l_{1}+1}{2}}-{\bar{\kappa }}_{3}V_{3}^{\frac{\frac{1}{l_{2}}+1}{2}}\leq -{\bar{\kappa }}_{2}V_{3}^{{\bar{l}}_{1}}-{\bar{\kappa }}_{3}V_{3}^{{\bar{l}}_{2}}$$
  where ${\bar{l}}_{1}=\frac{l_{1}+1}{2}$ and ${\bar{l}}_{2}=\frac{l_{2}+1}{2}$.
- When |s|<1:
  For $r_{1}=1/l_{1}<1$ and $r_{2}=l_{2}<1$, the inequality similarly holds:
  $$-{\bar{\kappa }}_{2}V_{3}^{\frac{\frac{1}{l_{1}}+1}{2}}-{\bar{\kappa }}_{3}V_{3}^{\frac{l_{2}+1}{2}}\leq -{\bar{\kappa }}_{2}V_{3}^{{\bar{l}}_{1}}-{\bar{\kappa }}_{3}V_{3}^{{\bar{l}}_{2}}$$

Combining both cases, we express ${\dot{V}}_{3}$ as:

(35) $${\dot{V}}_{3}\leq -{\bar{\kappa }}_{2}V_{3}^{{\bar{l}}_{1}}-{\bar{\kappa }}_{3}V_{3}^{{\bar{l}}_{2}}+\ell$$

Equation (35) matches the fixed-time convergence form described in [35], confirming that the sliding variable *s* converges to within υ in a fixed time.

**Case 2:** When |s|<υ

For |s|<υ, ${\dot{\hat{\kappa }}}_{1}=-G^{-1}{\hat{\kappa }}_{1}\frac{\left|s\right|}{\upsilon }$. Thus, ${\dot{V}}_{2}$ becomes

(36) $$\begin{array}{cc} {\dot{V}}_{2} & =s\left[A\left(\frac{D}{I_{z}}-{\hat{\kappa }}_{1}\mathrm{sgn}\left(s\right)-\frac{1}{N\left(s\right)}\left(\kappa_{2}\mathrm{si}g^{r_{1}}\left(s\right)+\kappa_{3}\mathrm{si}g^{r_{2}}\left(s\right)\right)\right)\right]+\rho \left(\kappa_{1}-{\hat{\kappa }}_{1}\right)\frac{{\hat{\kappa }}_{1}\left|s\right|}{G\upsilon }\\ \; & =sA\frac{D}{I_{z}}-A{\hat{\kappa }}_{1}\left|s\right|+\rho \left(\kappa_{1}-{\hat{\kappa }}_{1}\right)\frac{{\hat{\kappa }}_{1}\left|s\right|}{G\upsilon }-\frac{A}{N\left(s\right)}\left(\kappa_{2}{\left|s\right|}^{r_{1}+1}+\kappa_{3}{\left|s\right|}^{r_{2}+1}\right)\\ \; & \leq \left|s\right|A\left(\frac{\bar{D}}{I_{z}}-{\hat{\kappa }}_{1}\right)+\rho \left(\kappa_{1}-{\hat{\kappa }}_{1}\right)\frac{{\hat{\kappa }}_{1}\left|s\right|}{G\upsilon }-\frac{A}{N\left(s\right)}\left(\kappa_{2}{\left|s\right|}^{r_{1}+1}+\kappa_{3}{\left|s\right|}^{r_{2}+1}\right)\\ \; & \leq \left|s\right|\left(\kappa_{1}-{\hat{\kappa }}_{1}\right)\left(A+\rho \frac{{\hat{\kappa }}_{1}}{G\upsilon }\right)-\frac{A}{N\left(s\right)}\left(\kappa_{2}{\left|s\right|}^{r_{1}+1}+\kappa_{3}{\left|s\right|}^{r_{2}+1}\right)\\ \; & \leq \left|s\right|\left(\kappa_{1}-{\hat{\kappa }}_{1}\right)\left(A+\rho \frac{{\hat{\kappa }}_{1}}{G\upsilon }\right)-A\left(\kappa_{2}{\left|s\right|}^{r_{1}+1}+\kappa_{3}{\left|s\right|}^{r_{2}+1}\right) \end{array}$$

When |s|≥υ, |s| decreases until it enters the region |s|<υ. We can derive an upper bound for |s| by considering:

(37) $$0.5s^{2}\leq V_{2}\leq 0.5s^{2}+0.5\rho {\tilde{\kappa }}_{1}^{2}$$

For s<υ, the upper bound of $V_{2}$ is

(38) $$V_{2}\leq 0.5\upsilon^{2}+0.5\rho {\tilde{\kappa }}_{1}^{2}$$

This ensures the existence of $\bar{\kappa }$, representing the maximum value of $\rho {\tilde{\kappa }}_{1}^{2}$, such that:

(39) $$V_{2}\leq 0.5\upsilon^{2}+0.5\bar{\kappa }$$

Consequently, the upper bound for |s| when |s|<υ is

(40) $$\left|s\right|\leq \sqrt{\upsilon^{2}+\bar{\kappa }}$$

Thus, the closed-loop system is uniformly ultimately bounded, verifying the stability of the proposed control approach.

### 3.3. Lower-Level Controller

To effectively implement the additional yaw moment generated by the proposed adaptive NFTSMC method, allocating the torque optimally across the four wheels is crucial. Various distribution strategies, such as average distribution [36] and optimal allocation based on dynamic vertical load [2], have been widely employed to improve vehicle stability and handling in response to changing driving conditions.

When a vehicle turns, significant variations exist in the load distribution across the wheels owing to lateral and longitudinal forces. These variations are attributable to factors, including the road surface friction coefficient, which affects the available tire-road contact force. This study adopts a torque distribution strategy that considers the dynamic vertical load ratio on each wheel. As the vertical load on each wheel increases or decreases, the corresponding torque allocation is adjusted proportionally, enhancing the efficiency of longitudinal force utilization.

The torque allocation is achieved by distributing the longitudinal force obtained from the upper-level controller according to the vertical load ratio on each wheel. By considering the load ratio, each wheel’s available traction is optimized, resulting in more precise handling and improved vehicle stability during maneuvers.

To derive the relationship between the longitudinal force and yaw moment generated by each tire, the following equations specify the driving torques for the four wheels based on their correlation with the longitudinal forces:

(41) $$\left\{\begin{array}{c} T_{dfl}=\frac{F_{xfl}}{R}=\frac{F_{zfl}}{F_{z}R}\cdot \frac{\Delta M_{z}}{l_{f}\sin\delta_{f}-\frac{d_{f}}{2}\cos\delta_{f}}\\ T_{dfr}=\frac{F_{xfr}}{R}=\frac{F_{zfr}}{F_{z}R}\cdot \frac{\Delta M_{z}}{l_{f}\sin\delta_{f}+\frac{d_{f}}{2}\cos\delta_{f}}\\ T_{drl}=\frac{F_{xrl}}{R}=-\frac{F_{zrl}}{F_{z}R}\cdot \frac{\Delta M_{z}}{2d_{r}}\\ T_{drr}=\frac{F_{xrr}}{R}=\frac{F_{zrr}}{F_{z}R}\cdot \frac{\Delta M_{z}}{2d_{r}} \end{array}\right.$$

Here, $T_{dfl},T_{dfr},T_{drl},$ and $T_{drr}$ represent the driving torques for the front-left, front-right, rear-left, and rear-right wheels, respectively. Here, $F_{zfl},F_{zfr},F_{zrl},$ and $F_{zrr}$ are the vertical loads on each wheel, while $F_{z}$ denotes the total vertical load. The additional yaw moment, $\Delta M_{z}$, is divided based on the distances between the wheels and the vehicle’s center of gravity, denoted by *a*, $d_{f}$, and $d_{r}$.

This allocation strategy enables each wheel to effectively generate the yaw moment, enhancing cornering performance and stability, particularly on surfaces with variable traction conditions. By dynamically adjusting torque distribution according to vertical load ratios, the proposed lower-level controller ensures that the yaw moment is applied with maximum efficiency. This supports the overall stability and maneuverability of the vehicle in real-time driving scenarios.

## 4. Simulation Results and Analysis

### 4.1. Simulation Environment

The tests were conducted using the co-simulation of Matlab/Simulink R2021b and CarSim 2022.0 on an offline simulation platform. A B-Class hatchback vehicle model in CarSim, equipped with a complete 4WID chassis dynamic system, was chosen as the plant model. This model was chosen because it represents a widely utilized and versatile class of vehicles, balancing size, weight, and performance characteristics. Such a choice ensures that the experimental results are broadly relevant to typical driving conditions encountered during daily use, thereby enhancing the practical applicability of our findings. Additionally, the B-Class hatchback provides a representative platform for testing advanced control strategies due to the moderate complexity of its dynamics. Its characteristics align well with vehicles commonly used in similar research, facilitating meaningful comparisons and benchmarking with existing studies. This model includes longitudinal, lateral, and yaw motions, along with the rotational dynamics of all four wheels, while vertical motion is excluded. Load transfer was considered within the model to enhance simulation accuracy. Table 1 illustrates the parameters of the vehicle model used in the tests.

**Figure 4:**
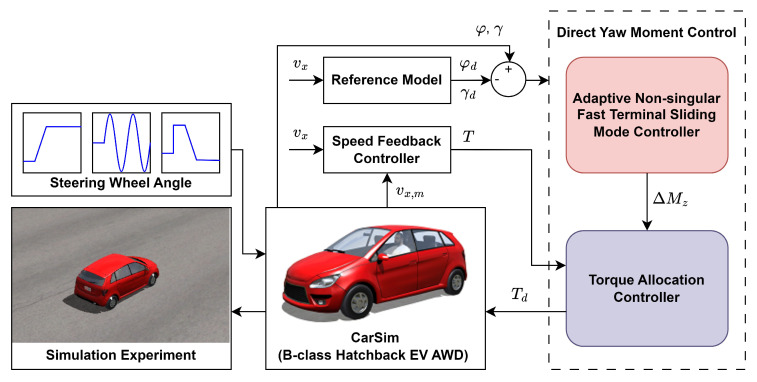
System diagramof the test flow in simulation.

Figure 4 shows the overall flow of the simulation tests. To ensure lateral driving stability at the specified speed, the vehicle must precisely track the desired yaw rate on a flat, dry asphalt road. The proposed upper-level controller calculates the required additional yaw moment. The lower-level controller distributes it as driving torque to each of the four wheels, thereby achieving yaw rate tracking. For fair comparisons, the torque allocation within the lower-level controller remained identical across all experiments.

**Figure 5:**
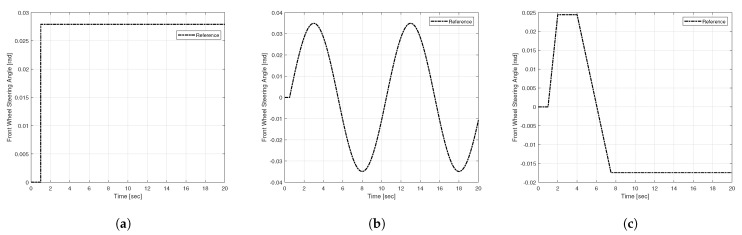
Front wheel steering angle for test scenario: (**a**) Case1—step, (**b**) Case2—sine wave, (**c**) Case3—fish-hook.

Finally, simulations we performed for the three standard test maneuvers, step input, sine input, and fish-hook input, as shown in Figure 5 [37]. This study compares the proposed DYC controller with existing control techniques, without control, SMC, NFTSMC, to demonstrate the effectiveness of the proposed method. An analysis of yaw rate tracking performance based on RMS (root mean squared) and peak error shows that the proposed method accurately tracked the ideal yaw rate under various driving conditions. All controllers were tested under identical initial conditions and environment settings, and their design specifications were provided accordingly.

The additional yaw moment control law of SMC is designed as:

(42) $$\Delta M_{z}=I_{z}\left[\dot{\gamma_{d}}-c\dot{e}-\eta_{1}\mathrm{sgn}\left(s\right)-\eta_{2}s\right]-F$$

where $s=\dot{e}+ce$ represents the linear sliding mode surface, c>0, $\eta_{1}>0$, and $\eta_{2}>0$.

The additional yaw moment control law of NFTSMC is expressed as:

(43) $$\Delta M_{z}=I_{z}\left[\dot{\gamma_{d}}-\frac{{\left|\dot{e}\right|}^{2-q}}{q\lambda_{2}}\left(1+p\lambda_{1}{\left|e\right|}^{p-1}\right)\mathrm{sgn}\left(\dot{e}\right)-\eta_{1}\mathrm{sgn}\left(s\right)-\eta_{2}s\right]-F$$

where *s* is defined as Equation (11), p>q, 1<q<2, $\eta_{1}>0$, and $\eta_{2}>0$.

### 4.2. Simulation Results

#### 4.2.1. Case 1—Step Input Maneuver

**Figure 6:**
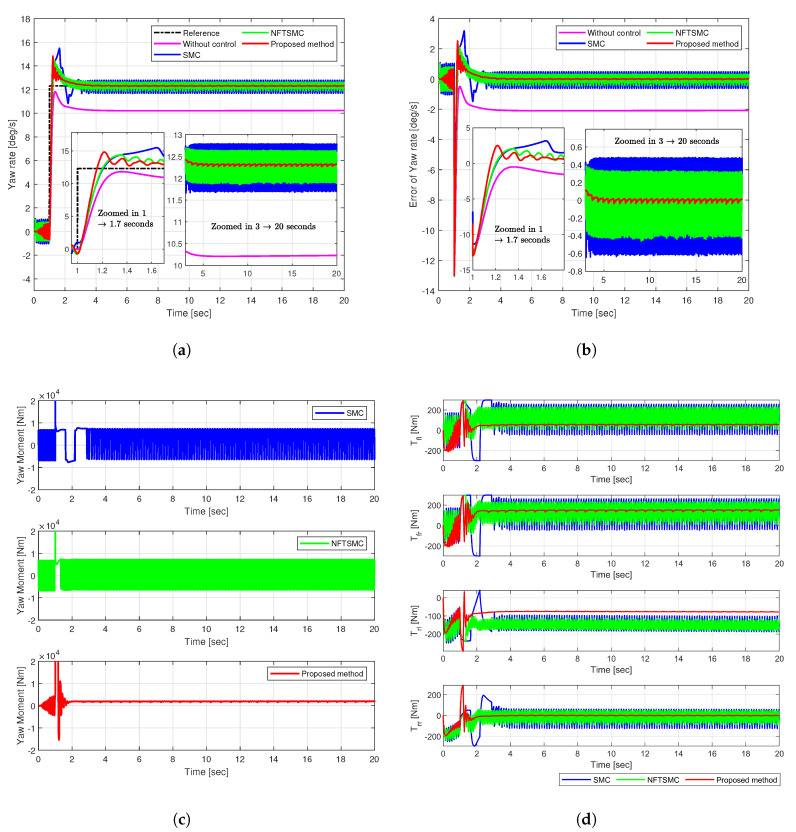
Simulation results of comparison in Case 1: (**a**) Yaw rate, (**b**) Tracking error, (**c**) Yaw moment, (**d**) Torque.

**Figure 7:**
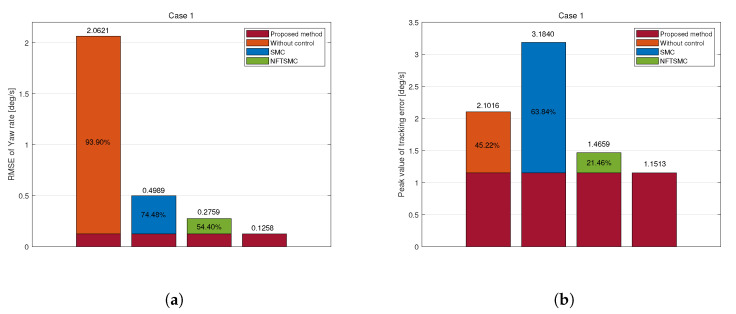
Tracking error of yaw rate in Case 1: (**a**) RMSE, (**b**) Peak.

The step input maneuver involved applying a step input to the front steering angle at a specified vehicle speed, simulating a situation where the steering angle rapidly increased, akin to an emergency collision avoidance scenario. The vehicle speed was set at 72 km/h. Figure 6 and Figure 7 illustrate the simulation results. The driving stability of the vehicle was evaluated based on the yaw rate at the vehicle’s center of gravity.

Figure 6a,b illustrates the yaw rate tracking performance and yaw rate error. Regarding the uncontrolled vehicle, the yaw rate stabilized at approximately 10.2 deg/s after 2 s, resulting in a loss of driving stability. The existing SMC, NFTSMC, and the proposed method ultimately achieved stability and fulfilled the control objectives. To compare control performance, we examined results during the transient state (from 1.1 to 1.7 s) and the steady state (from 3 to 20 s). The proposed controller exhibited a faster response rate, achieving system stability more rapidly than the traditional SMC and NFTSMC approaches. Additionally, while all three methods exhibited oscillations in the steady state, the proposed method yielded the smallest oscillation frequency and magnitude. This improvement was attributable to the adaptive fast-reaching control law, which enhances convergence speed. Moreover, the adaptive mechanism adjusts the sliding gain in real-time to reflect changes in system state and disturbances, increasing robustness against disturbances and modeling uncertainties and enhancing driving stability.

The proposed method records the lowest values for RMS and peak tracking errors, measuring 0.1258 deg/s and 1.1513 deg/s, respectively (Figure 7). Compared to those of the uncontrolled, SMC, and NFTSMC methods, the RMS errors were reduced by 93.90%, 74.48%, and 54.40%, while the peak errors were decreased by 45.22%, 63.84%, and 21.46%. These results reflect the superiority of the proposed control method, demonstrating that the proposed DYC control strategy enhances stability and accuracy.

The yaw moment control input shows significant chattering in the existing SMC and NFTSMC methods, with values rising sharply to approximately 13,000 Nm (Figure 6c). This indicates sensitivity to disturbances before reaching the sliding surface, leading to abrupt and excessive changes. However, the proposed method demonstrates insignificant chattering between 1 and 1.7 s, stabilizing at approximately 100 Nm, and thus, enhancing vehicle stability.

Existing methods exhibit significant chattering phenomena in the yaw moment control signal (Figure 6c). The abrupt increase in control signal during specific intervals may be challenging for the actual plant’s actuator to implement effectively. To address this issue, the proposed adaptive mechanism automatically adjusts the adaptation gain ${\hat{\kappa }}_{1}$, effectively suppressing chattering. This significantly reduces chattering in the yaw moment and the torque distributed to each wheel.

The simulation results regarding the torque allocated to each wheel from the yaw moment further validate the effectiveness of the proposed method. The pronounced chattering effect of the yaw moment in traditional SMC and NFTSMC causes significant torque fluctuations in each wheel. In contrast, the proposed method substantially reduces chattering yaw moment chattering, resulting in smoother torque distribution across all wheels and enhancing driving safety.

#### 4.2.2. Case 2—Sine Input Maneuver

**Figure 8:**
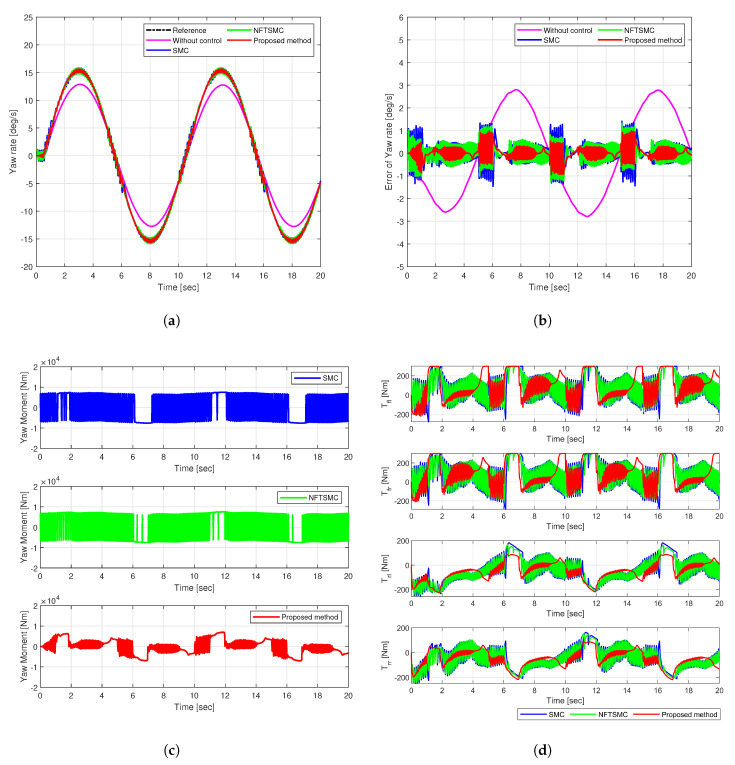
Simulation results of comparison in Case 2: (**a**) Yaw rate, (**b**) Tracking error, (**c**) Yaw moment, (**d**) Torque.

**Figure 9:**
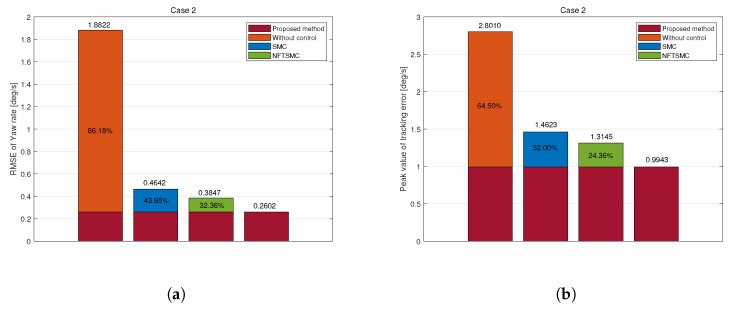
Tracking error of yaw rate in Case 2: (**a**) RMSE, (**b**) Peak.

The sine input maneuver simulates continuous directional changes at a constant speed of 72 km/h by applying a sinusoidal input to the vehicle’s steering angle. Figure 8 and Figure 9 illustrate the simulation results, indicating the yaw rate, tracking error, yaw moment, and the torque distributed to each wheel.

SMC, NFTSMC, and the proposed method successfully follow the reference trajectory, as shown in Figure 8a. However, the uncontrolled vehicle fails to reach the reference value, resulting in understeering. The yaw rate tracking error shows that the error of the proposed method (in red) remains the most insignificant among other methods, as shown in Figure 8b. The RMS and peak values of the yaw rate tracking error for the proposed method were 0.2602 deg/s and 0.9943 deg/s, respectively. Compared to the uncontrolled, SMC, and NFTSMC cases, the proposed method achieved reductions in RMSE of 86.18%, 43.95%, and 32.36%. Moreover, peak values decreased by 64.50%, 32.00%, and 24.36%, respectively. These results demonstrate the superior tracking accuracy and robustness of the proposed control method against disturbances and uncertainties.

Figure 8c illustrates the additional yaw moment signals. The existing SMC and NFTSMC methods exhibit substantial chattering in yaw moment control input, reaching approximately 13,500 Nm. However, the proposed method effectively suppressed chattering, significantly reducing its frequency and magnitude to a maximum of 7700 Nm. This reduction in yaw moment chattering decreased chattering in the torque distributed to each wheel, as shown in Figure 8d.

The excellent stability and accuracy of the proposed method stem from the adaptive control mechanism, which adjusts the sliding gain by reflecting real-time system state and disturbance changes. This feature enhanced robustness against disturbances and uncertainties. Moreover, it enhanced driving stability while accelerating convergence speed and control accuracy. The fast-reaching control law facilitated rapid convergence to the sliding surface, minimizing the influence of disturbances and reducing oscillations in the yaw moment and torque signals.

#### 4.2.3. Case 3—Fish-Hook Input Maneuver

**Figure 10:**
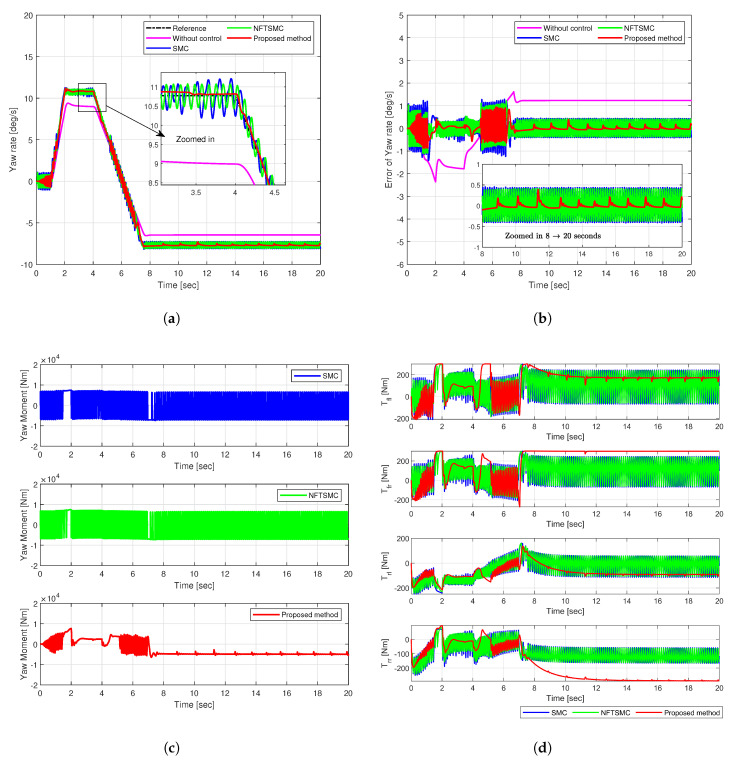
Simulation results of comparison in Case 3: (**a**) Yaw rate, (**b**) Tracking error, (**c**) Yaw moment, (**d**) Torque.

**Figure 11:**
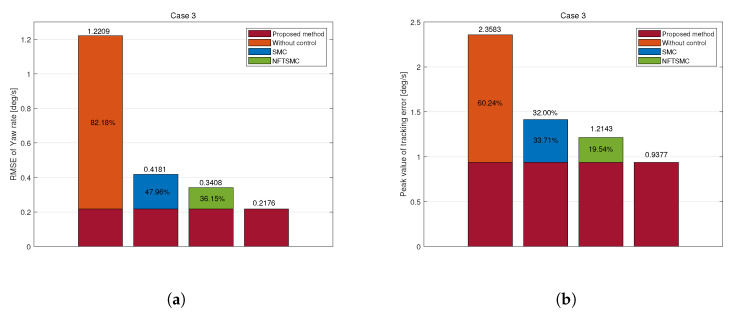
Tracking error of yaw rate in Case 3: (**a**) RMSE, (**b**) Peak.

The fish-hook input maneuver simulates a situation in which the vehicle performs continuous, abrupt steering maneuvers at 72 km/h under a fish-hook steering angle input. Figure 10 and Figure 11 illustrate the simulation results, indicating the yaw rate, tracking error, yaw moment, and torque distribution across each wheel.

SMC, NFTSMC, and the proposed method closely track the reference yaw rate, as shown in Figure 10a. However, the uncontrolled vehicle fails to reach the reference value, resulting in an understeer phenomenon. Among the controlled approaches, the proposed control method demonstrates the fastest and most precise tracking, maintaining the smallest yaw rate error, as shown in Figure 10b.

The proposed method achieved RMS and peak values for yaw rate tracking errors of 0.2176 deg/s and 0.9377 deg/s, respectively. Compared to those of the uncontrolled, SMC, and NFTSMC methods, RMS reductions were 82.18%, 47.66%, and 63.15%, and peak value reductions were 60.24%, 33.71%, and 19.54%, respectively.

The proposed yaw moment control input minimizes yaw rate tracking errors. Moreover, it significantly reduced chattering compared to conventional SMC and NFTSMC methods, which produce high chattering amplitudes of 13,500 Nm, as shown in Figure 10c. This reduction in yaw moment chattering translates to torque distribution across each wheel, as shown in Figure 10d.

#### 4.2.4. Overall Performance Evaluation

This simulation comprehensively evaluated the performance of the proposed method across step, sinusoidal, and fish-hook input scenarios. In each scenario, the proposed method effectively controlled abrupt changes in yaw moment during the vehicle’s steering process and dynamically distributed longitudinal tire forces. This significantly enhanced tracking performance and driving stability. Consequently, the vehicle accurately followed the desired yaw rate, effectively reducing the RMS and peak values of the yaw rate error. This prevented potential safety-compromising situations, such as understeer, that can arise in uncontrolled conditions.

**Figure 12:**
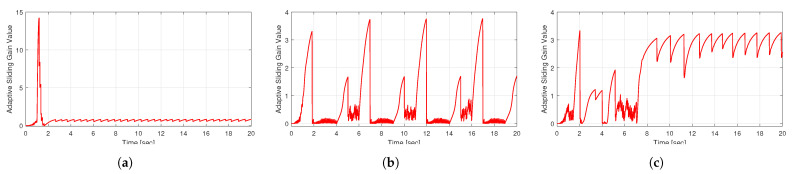
Adaptive sliding gains of the proposed control method: (**a**) Case1, (**b**) Case2, (**c**) Case3.

These results highlight the superior performance of the proposed method in terms of tracking accuracy, convergence speed, and driving stability. Through the proposed adaptive-fast reaching control law, the system quickly reaches the sliding surface, minimizing the influence of external disturbances. Moreover, its adaptive gain adjustment through an adaptive mechanism suppresses chattering, significantly enhancing system stability. Figure 12 illustrates the switching gain adjustments across each scenario. Table 2 and Table 3 illustrate the RMS and peak values of the yaw rate tracking error for each scenario, respectively.

## 5. Conclusions

This study introduced a novel adaptive NFTSMC-based DYC method to improve the driving stability of 4WID electric vehicles. The proposed approach offers robust performance against disturbances and modeling uncertainties through adaptive control. Moreover, the fast-reaching control law ensures rapid system convergence, achieving high tracking accuracy and driving stability. Simulation results demonstrated that the proposed method effectively reduces RMS and peak errors and suppresses chattering in yaw moment and torque signals under various steering input scenarios, exhibiting superior performance compared to conventional control methods. These findings highlight the practical applicability of the proposed DYC method for stable and precise vehicle motion control in electric vehicles. Future work will involve experimental validation on real vehicles and exploration of potential extensions of this control approach.

## Figures and Tables

**Table 1:** Specification of vehicle model.

Specification	Unit	Symbol	Value
Vehicle Mass	kg	*m*	1134
Wheelbase Length	mm	*L*	2600
CoG to Front Axle Distance	mm	$l_{f}$	1040
CoG to Rear Axle Distance	mm	$l_{r}$	1560
Front Track Width	mm	$d_{f}$	1485
Rear Track Width	mm	$d_{r}$	1485
Wheel Radius	mm	*R*	1485
Yaw Moment of Inertia	$\mathrm{kg}\cdot m^{2}$	$I_{z}$	1343.1

**Table 2:** RMS of the yaw rate tracking errors.

Method	Case1		Case2		Case3	
	$e_{\mathrm{rms}}$	**Reduction Rate**	$e_{\mathrm{rms}}$	**Reduction Rate**	$e_{\mathrm{rms}}$	**Reduction Rate**
Propose method	**0.1258**	−	**0.2602**	−	**0.2176**	−
Without control	2.0621	−93.90%	1.8822	−86.18%	1.2209	−82.18%
SMC	0.4989	−74.48%	0.4642	−43.95%	0.4181	−47.96%
NFTSMC	0.2759	−54.40%	0.3847	−32.36%	0.3408	−36.15%

**Table 3:** Peak value of the yaw rate tracking errors.

Method	Case1		Case2		Case3	
	$\left|e_{\max}\right|$	**Reduction Rate**	$\left|e_{\max}\right|$	**Reduction Rate**	$\left|e_{\max}\right|$	**Reduction Rate**
Propose method	**1.1513**	−	**0.9943**	−	**0.9377**	−
Without control	2.1016	−45.22%	2.8010	−64.50%	2.3583	−60.24%
SMC	3.1840	−63.84%	1.4623	−32.00%	1.4146	−33.71%
NFTSMC	1.4659	−21.46%	1.3145	−24.36%	1.2143	−19.54%

## Data Availability

Data are contained within the article.
